# A Novel Method for Assessing Cerebral Edema, Infarcted Zone and Blood-Brain Barrier Breakdown in a Single Post-stroke Rodent Brain

**DOI:** 10.3389/fnins.2019.01105

**Published:** 2019-10-16

**Authors:** Ruslan Kuts, Dmitry Frank, Benjamin F. Gruenbaum, Julia Grinshpun, Israel Melamed, Boris Knyazer, Oleg Tarabrin, Vladislav Zvenigorodsky, Ilan Shelef, Alexander Zlotnik, Matthew Boyko

**Affiliations:** ^1^Division of Anesthesiology and Critical Care, Soroka University Medical Center and the Faculty of Health Sciences, Ben-Gurion University of the Negev, Beersheba, Israel; ^2^Department of Anesthesiology, Yale University School of Medicine, New Haven, CT, United States; ^3^Department of Neurosurgery, Soroka University Medical Center and the Faculty of Health Sciences, Ben-Gurion University of the Negev, Beersheba, Israel; ^4^Department of Ophthalmology, Soroka University Medical Center and the Faculty of Health Sciences, Ben-Gurion University of the Negev, Beersheba, Israel; ^5^Department of Anesthesiology and Intensive Care with Postgraduate Education, Odessa National Medical University, Odessa, Ukraine; ^6^Department of Radiology, Soroka University Medical Center and the Faculty of Health Sciences, Ben-Gurion University of the Negev, Beersheba, Israel

**Keywords:** stroke, middle cerebral artery occlusion, rodent, model, methods

## Abstract

Stroke is a major cause of global morbidity and mortality. Middle cerebral artery occlusion (MCAO) has historically been the most common animal model of simulating ischemic stroke. The extent of neurological injury after MCAO is typically measured by cerebral edema, infarct zone, and blood-brain barrier (BBB) permeability. A significant limitation of these methods is that separate sets of brains must be used for each measurement. Here we examine an alternative method of measuring cerebral edema, infarct zone and BBB permeability following MCAO in the same set of brain samples. Ninety-six rats were randomly divided into three experimental groups. Group 1 (*n* = 27) was used for the evaluation of infarct zone and brain edema in rats post-MCAO (*n* = 17) vs. sham-operated controls (*n* = 10). Group 2 (*n* = 27) was used for the evaluation of BBB breakdown in rats post-MCAO (*n* = 15) vs. sham-operated controls (*n* = 10). In Group 3 (*n* = 42), all three parameters were measured in the same set of brain slices in rats post-MCAO (*n* = 26) vs. sham-operated controls (*n* = 16). The effect of Evans blue on the accuracy of measuring infarct zone by 2,3,5-triphenyltetrazolium chloride (TTC) staining was determined by measuring infarct zone with and without an applied blue filter. The effects of various concentrations of TTC (0, 0.05, 0.35, 0.5, 1, and 2%) on the accuracy of measuring BBB permeability was also assessed. There was an increase in infarct volume (*p* < 0.01), brain edema (*p* < 0.01) and BBB breakdown (*p* < 0.01) in rats following MCAO compared to sham-operated controls, whether measured separately or together in the same set of brain samples. Evans blue had an effect on measuring infarct volume that was minimized by the application of a blue filter on scanned brain slices. There was no difference in the Evans blue extravasation index for the brain tissue samples without TTC compared to brain tissue samples incubated in TTC. Our results demonstrate that measuring cerebral edema, infarct zone and BBB permeability following MCAO can accurately be measured in the same set of brain samples.

## Introduction

Stroke is a leading cause of death and disability (Warlow, 1998; Lloyd-Jones et al., 2009; Feigin et al., 2016, 2017). Over 16 million people worldwide suffer from stroke each year with approximately one-third resulting in death and another third developing permanent disability (Bejot et al., 2016; Thrift et al., 2017). Stroke is also a source of substantial economic burden, with an estimated annual cost in the United States and European Union of $34 billion (Benjamin et al., 2017) and €45 billion (Wilkins et al., 2017), respectively. Animal models are necessary to better understand the pathophysiology of stroke and to advance the development of new therapies targeted at reducing and repairing neurological damage.

Ischemic stroke accounts for over 80% of all strokes and results from an occlusion of a major cerebral artery, most often the middle cerebral artery or one of its branches (Lloyd-Jones et al., 2009). Consequently, middle cerebral artery occlusion (MCAO) has historically been the most common animal model of simulating ischemic stroke (Waltz et al., 1966; Hudgins and Garcia, 1970; Albanese et al., 1980; Tamura et al., 1981; Shigeno et al., 1985; Aspey et al., 1998). When determining the extent of neurological injury in the MCAO model, measured outcomes include cerebral edema (O’Brien et al., 1974; Chen et al., 2006; Durukan and Tatlisumak, 2007), infarct zone (Wang-Fischer, 2008; Liu et al., 2009) and blood-brain barrier (BBB) permeability (Belayev et al., 1996; Sifat et al., 2017; Jiang et al., 2018). Following MCAO, the most popular techniques for measuring brain edema are drying (Chen et al., 2006) or calculating hemispheric volumes (Boyko et al., 2010, 2019b). The infarct zone is mostly determined by a 2,3,5-triphenyltetrazolium chloride (TTC) staining method (Liu et al., 2009) that differentiates between infarcted and viable tissue (Joshi et al., 2004). To measure the BBB breakdown, a spectrometry technique using Evans blue staining is most commonly used (Belayev et al., 1996).

A significant limitation of these methods to assess neurological injury after MCAO is that separate sets of brains must be used for each measurement. Thus, in order to obtain accurate and statistically reliable results, researchers tend to use large number of brain samples resulting in a large number of euthanized animals. Therefore, there is a significant ethical and economical benefit if all three of these parameters could be measured post-MCAO in a single set of rodent brains.

There is evidence in the literature that several parameters can be used on the same brain sample. For example, a combination of TTC staining and immunofluorescent staining methods (Li et al., 2018) as well as other molecular and biochemical analyses after TTC staining (Kramer et al., 2010) have been described. Calculating brain hemisphere volumes to assess brain edema has been performed in our laboratory together with TTC staining to calculate infarct zone using the same brain set (Boyko et al., 2019b). However, measuring BBB permeability in the same set of brain samples remains a challenge and has yet to be described in the literature. This is likely due to the possible influence of one staining technique on the accuracy of a subsequent staining method (Evans blue and TTC, for example). The purpose of the present study was to examine an alternative method of measuring all three parameters following MCAO in the same set of brain samples: cerebral edema, infarct zone and BBB permeability.

For this purpose, we combined the following protocols in a single set of rat brains: TTC staining for measuring infarct zone, calculating hemispheric volumes to measure cerebral edema, and a spectrometry technique using Evans blue staining for evaluating BBB breakdown. We evaluated the effects of TTC staining on the accuracy of BBB permeability measurements, as well as the effects of Evans blue staining on the accuracy of infarct zone measurements. We further compared histologic techniques of assessing neurological injury post-MCAO to Magnetic Resonance Imaging (MRI) techniques. This new approach may serve as an effective, economical, and ethically favorable model for measuring neurological injury after MCAO.

## Materials and Methods

The experiments were conducted in accordance with the recommendations of the Declarations of Helsinki and Tokyo and the Guidelines for the Use of Experimental Animals of the European Community. The experiments were approved by the Animal Care Committee of Ben-Gurion University of the Negev, Israel.

### Animals

The experiments were carried out on a total of 96 Sprague-Dawley rats (Harlan Laboratories, Israel) with no overt pathology, weighing between 300 and 400 g each. Purina Chow and water were available *ad libitum*. Rats were maintained in a 12:12 h light–dark cycle and at constant temperature (22°C ± 1°C).

### Experimental Design

Ninety-six rats were randomly divided into three experimental groups. Group 1 (*n* = 27) was used for the evaluation of infarct zone and brain edema using original techniques previously described in the literature (Kaplan et al., 1991; Joshi et al., 2004; Boyko et al., 2011a, 2013). These rats were subjected to either MCAO (*n* = 17, of which 11 remained after exclusion criteria) or used as a sham-operated control group without MCAO (*n* = 10). TTC staining was used 24 h after MCAO to measure infarct zone and cerebral edema was measured by calculating hemispheric volumes. Group 2 (*n* = 27) was used for the evaluation of BBB breakdown using the original technique previously described (Boyko et al., 2012). These rats were subjected to either MCAO (*n* = 17, of which 12 remained after exclusion criteria) or used as a sham-operated control group without MCAO (*n* = 10). BBB disruption was determined 24 h after MCAO by a spectrometry technique using an intravenous injection of 2% Evans blue in saline (4 ml/kg). Group 3 (*n* = 42) was used to assess the feasibility of a new technique measuring all three parameters in the same set of brain slices: infarct zone by TTC staining, cerebral edema by calculating hemispheric volumes, and BBB breakdown by Evans blue staining. These rats were subjected to either MCAO (*n* = 26, of which 19 remained after exclusion criteria) or used as a sham-operated control group without MCAO (*n* = 16). The new technique for measuring all three parameters is described below. All rats underwent evaluation of neurological deficit 24 h after the operation.

### Surgery for MCAO Model of Stroke

The MCAO procedure was performed according to the method described by Zea Longa (Longa et al., 1989), modified by an internal carotid artery (ICA) approach (Boyko et al., 2010). This approach was chosen because it has been shown to produce lower variability in the infarct volume, better weight gain after surgery and reduced mortality (Boyko et al., 2010).

The operation was performed under aseptic conditions in accordance with accepted principles in animal surgeries. Rats were anesthetized with a mixture of isoflurane (4% for induction, 2% for surgery, 1.3% for maintenance) in 24% oxygen (2 l/min) without tracheotomy, and were allowed to breathe spontaneously. Core body temperature was maintained at 37°C throughout the procedure with a rectal temperature-regulated heating pad. Body temperature was kept constant between rats to minimize any effect of hypothermia or hyperthermia on neurological outcome and neurological injury. Physiological parameters, including mean arterial pressure, heart rate and O2 saturation of arterial blood, were monitored.

The right common carotid artery (CCA) was exposed through a midline neck incision and was carefully dissected from surrounding tissues, from its bifurcation to the base of the skull. The catheter was then inserted via the ICA to achieve MCAO. The thread was then fixed by tying a silk filament over the ICA immediately above the CCA bifurcation and proximal to filament insertion point. The purpose of this proximal ligation was to occlude the ICA while the additional distal ligation was used to reduce the bleeding around the filament and to secure it in place. The suture was left in place permanently and the incision was closed using surgical sutures. After this procedure, the anesthesia was discontinued, and rats returned to their cages for recovery. The duration of the entire surgery was approximately 25–30 min. There were no differences in the time allotted for anesthesia between groups in order to control the effects of isoflurane, pO2, or pCO2. Rats that died within 24 h of the MCAO procedure were excluded from this study.

### Measurement of Neurological Deficit

An observer, who was blinded to the surgical procedure, tested the animals for neurological deficits 24 h after MCAO (Menzies et al., 1992). This scoring method was used as an exclusion criterion to identify and exclude rats that did not develop neurological deficits following MCAO. This exclusion criterion was to control for anatomic variations in rats’ middle cerebral artery branches (Rubino and Young, 1988; Fox et al., 1993; Zhao et al., 2008). Motor deficits were graded on a cumulative scale from 0 to 4. A score of 0 was given for no visible neurological deficits; a score of 1 was given for forelimb flexion; a score of 2 was given for contralateral weak forelimb grip (the operator places the animal on an absorbent pad and gently pulls the tail); a score of 3 was given for circling to the paretic side only when pulled by the tail (the animal was allowed to move about freely on the absorbent pad); and a score of 4 was given for spontaneous circling (Boyko et al., 2011a).

### Measurement of Brain Infarct Volume (Original Technique)

In order to measure the extent of brain edema in group 1, the TTC staining method was performed 24 h after the operation, as previously described (Kuts et al., 2019). The rats from each experiment subgroup were euthanized by inspiration of high CO2 and were decapitated. Their brains were quickly isolated and sectioned into 6 coronal slices, each 2 mm of thickness. The set of slices from each brain was incubated for 30 min at 37°C in 0.05% TTC. Following staining, the slices were scanned with an optical scanner (Canon Cano Scan 4200F; resolution 1600 × 1600 dpi). The unstained areas of the fixed brain slices were defined as infarcted, as described in the literature (Kuts et al., 2019). The size of brain injury was measured by the National Institutes of Health ImageJ software 1.37v, calculated in arbitrary units (pixels) and expressed as a percentage of the normal areas in the contralateral unaffected hemisphere. The total size of infarction was obtained by numeric integration of the area of marked pallor, measured in six consecutive 2 mm coronal sections (Boyko et al., 2011b). In order to correct for the tissue swelling factor, the following formula was utilized: corrected infarct size = infarct size × contralateral hemisphere size/ipsilateral hemisphere size. Infarcted volume was expressed as a percentage of the total brain (Boyko et al., 2011c).

### Measurement of Brain Edema (Original Technique)

The extent of right cerebral hemisphere edema was measured 24 h after the operation in Group 1. The volumes of both hemispheres were calculated in the arbitrary units (pixels) from the summation of coronal slice areas using the National Institutes of Health ImageJ software 1.37v, after they were scanned with an optical scanner (Canon Cano Scan 4200F; resolution 1600 × 1600 dpi). Brain edema was expressed as a percentage of the normal areas in the contralateral unaffected hemisphere. The extent of swelling was calculated using the Kaplan method: extent of edema = (the volume of right hemisphere – the volume of left hemisphere)/the volume of left hemisphere (Boyko et al., 2019a).

### Measurement of BBB Disruption (Original Technique)

In order to measure the extent of BBB disruption in Group 2, a spectrometry technique using Evans blue staining was performed 24 h after the operation. Two percent Evans blue in saline (4 ml/kg) was administered intravenously through a cannulated tail vein as a blood-brain permeability tracer and was allowed to circulate for 60 min. To remove the intravascularly localized dye, the rats’ chests were opened, and the animals were perfused with cooled saline through the left ventricle at a pressure of 110 mm Hg until colorless perfusion fluid was obtained from the right atrium. Their brains were quickly isolated and sliced rostro caudally into serial 2-mm thick slices. Then the brain slices were divided into the left and right hemisphere and measurements of vascular permeability were made by comparing their weight with pre-weighed loci in the six slices. Each brain area was weighted and homogenized in 1 ml of 50% trichloroacetic acid (weight/volume) and was centrifuged at 10,000 × *g* for 20 min and the supernatant was diluted 1:3 with 96% ethanol. A fluorescence detector (model Infinite 200 PRO multimode reader; Tecan, Männedorf Switzerland) was used at an excitation wavelength of 620 nm (bandwidth 10 nm) and an emission wavelength of 680 nm (bandwidth 10 nm). Calculations were based on external standards in the solvent (10 ± 500 ng/ml). Data were expressed as mean ± SD (in ng/g of protein) of extravasated Evans blue dye per gram of brain tissue (Boyko et al., 2012).

### New Technique for Measuring BBB Breakdown, Brain Infarct Volume and Brain Edema

**FIGURE 1:**
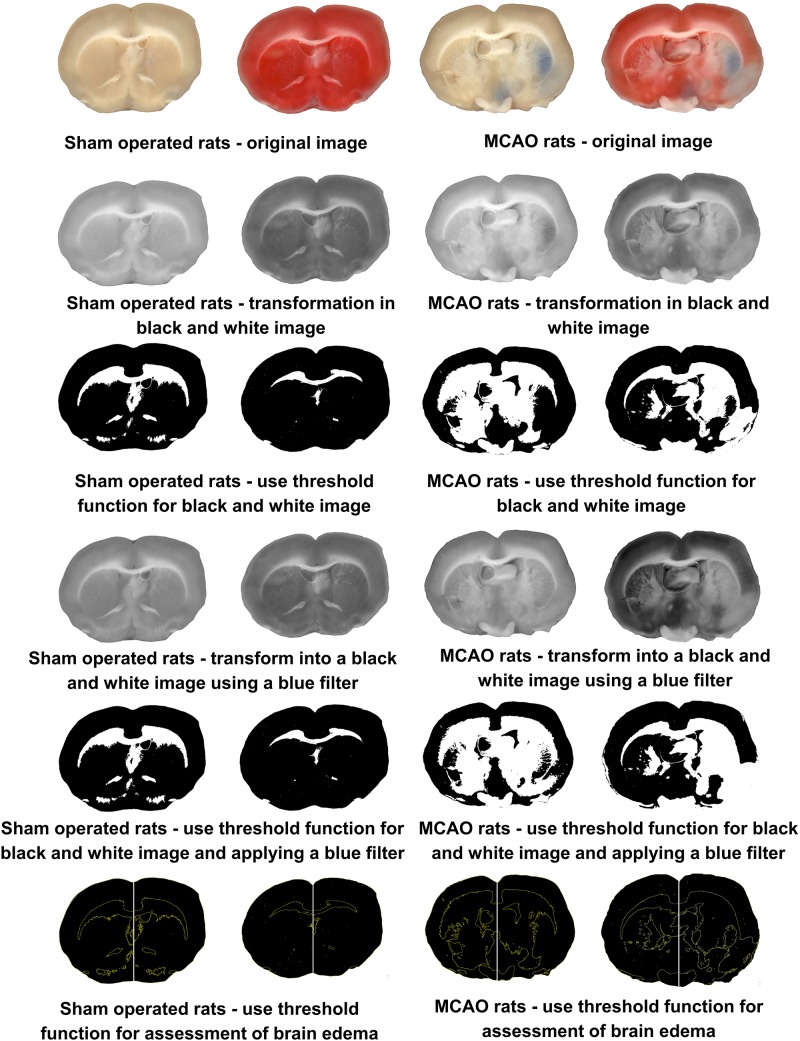
Histological brain scans from sham-operated rats and rats post-MCAO. Column one is a sham-operated brain slice without staining. Column two is a sham-operated brain slice with TTC staining. Column three is a post-MCAO brain slice with Evans blue staining. Column four is a post-MCAO brain slice with Evans blue and TTC staining.

For the rats in Group 3, 2% Evans blue in saline (4 ml/kg) was injected through the cannulated tail vein as a blood-brain permeability tracer and was allowed to circulate for 60 min. The rats’ chests were opened and the animals were perfused with cooled saline through the left ventricle. Their brains were quickly isolated and sectioned into 6 coronal slices, each 2 mm of thickness. The set of slices from each brain was then scanned (this scan was needed to later assess the effects of Evans blue staining on the accuracy of measuring infarct zone and is not required in the final protocol) and incubated for 30 min at 37°C in 0.05% TTC. The slices were again scanned with an optical scanner. Infarct zone and brain edema was measured with the National Institutes of Health ImageJ software 1.37v (Boyko et al., 2011c, 2019a). For these measurements, the computer program converts the scan into a black and white image and then uses a threshold function to mask and calculate the pixels that are either black or white (see Figure 1). In order to remove the effects of the Evans dye on this process, we added a blue filter using the Channel Mixer function (Image > Adjustments > Channel Mixer) from the Adobe Photoshop CS2 software program prior to calculating brain infarct zone and brain edema. After scanned, the following was performed in order to measure BBB disruption. The brain slice samples were divided into the left and right hemisphere and measurements of vascular permeability were made by comparing their weight with pre-weighed loci in the six slices. Each brain area was weighted and homogenized in 1 ml of 50% trichloroacetic acid (weight/volume) and was centrifuged at 10,000 × *g* for 20 min and the supernatant was diluted 1:3 with 96% ethanol. A fluorescence detector was used at an excitation wavelength of 620 nm (bandwidth 10 nm) and an emission wavelength of 680 nm (bandwidth 10 nm).

### Measurement of BBB Disruption by Brain Image Scanning

**FIGURE 2:**
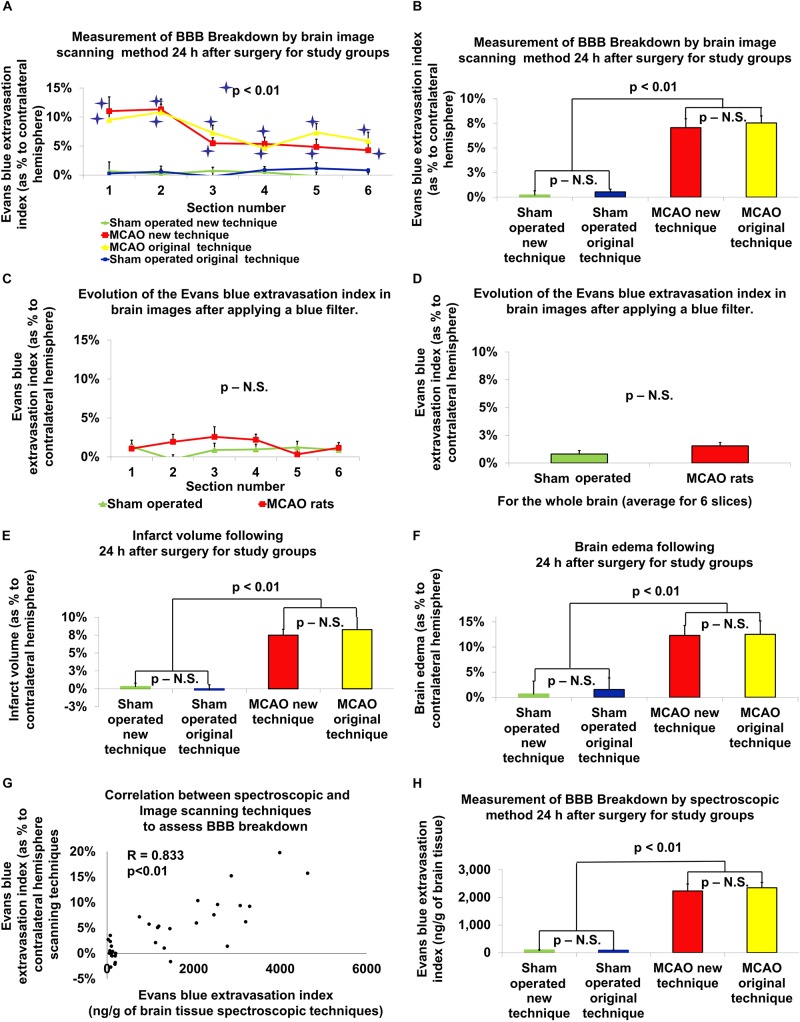
The new vs. original technique in evaluating cerebral edema, infarct zone and BBB permeability after MCAO. There was a significant difference in BBB breakdown measured by the brain image scanning method in all six brain slices in rats post-MCAO compared to sham-operated rats (*p* < 0.01; **A**; a significance asterisk indicates the difference between post-MCAO and sham-operated rats). The BBB breakdown, measured by the evaluation of the scanned brain slices, was significantly increased after MCAO compared to the sham-operated rats in both techniques (*p* < 0.01; **B**). After the application of the blue filter, there was no significant difference found between rats that underwent MCAO compared to sham-operated rats **(C,D)**. Both the new and old technique showed an increase in infarct volume (*p* < 0.01; **E**), brain edema (*p* < 0.01; **F**) and BBB breakdown (*p* < 0.01; **H**), following MCAO compared to sham-operated rats. There was no difference found between the old and new techniques. There was no difference found between the old and new techniques. There was a high correlation between the Evans blue extravasation index in brain tissue measured by the spectroscopic method and by the evaluation of scanned brain slices (*r* = 0.833, *p* < 0.01; **G**). The data is expressed as a mean percentage of the contralateral hemisphere ± SEM, or mean Evans blue extravasation index in ng/g of brain tissue ± SEM.

BBB disruption was also determined by a brain image scanning technique that has been described in the literature (Boyko et al., 2013) (see Figures 2A–D). Using the National Institutes of Health ImageJ software V1.63, the area of dye extravasation was measured using the previously described formula (Kumai et al., 2007): BBB disruption (as a percent): [left hemisphere – (right hemisphere – area stained blue)]/left hemisphere × 100. This was compared to the method of determining BBB disruption by spectrometry.

### Evaluating the Effect of Evans Blue Staining on the Accuracy of Measuring Infarct Zone

In order to evaluate the effects of Evans blue staining on the accuracy of measuring infarct zone, and the efficacy of the blue filter on scanned images to minimize this effect, the following procedure was performed. The six brain slices from Group 2 were scanned prior to being homogenized in 1 ml of 50% trichloroacetic acid during the measurement of BBB disruption via the original technique described above (Figure 1). The Evans blue extravasation index was then evaluated in these brain samples after the blue filter was applied.

### Evaluating the Effect of TTC Staining on the Accuracy of Measuring BBB Permeability

In order to assess the effect of TCC on the accuracy of measuring BBB permeability, samples not incubated in TTC were compared to brain tissue samples incubated in TTC solutions of various concentrations (0.05, 0.35, 0.5, 1, and 2%). Initially, it was a solution with 0% TTC concentrations on a standard 96-well plate, which was tested on a spectrograph. Then, TTC was added to each well based on the calculation of how much TTC is contained in brain samples when stained with solutions TTC of various concentrations. After each test, we increased the concentration until we reached 2%. BBB disruption was determined by spectrometry as described above.

### Comparison to MRI Techniques

In order to compare the original histologic to MRI imaging techniques, we analyzed data obtained from our previous work (Boyko et al., 2019b; Frank et al., 2019). The experimental procedure was carried out on a 3T MRI clinical scanner (Ingenia, Philips Medical Systems, Best, Netherlands), fitted out with a gradient system of 45 mT/m at 225 μs ramp time, using a commercial eight-channel receive-only wrist coil. MRI was performed 24 h after surgery for 17 post-MCAO and 19 sham-operated rats.

Preceding examination, a body restrainer was used to fix the rats for MRI. Animals, after horizontal placement into plastic holders, were given 4% isoflurane via inhalation from the anesthesia system, with 2% isoflurane mixed in 28% oxygen and 72% room air used for maintenance. A heating reel, filled with water and controlled thermostatically, was used to keep the body temperature of animals at 36.5–37.0°C. We measured pO2, pCO2, pH, arterial blood pressure, and the body core temperature before MRI and cannulated the tail artery and tail vein of rats.

T1 permeability studies were performed using a segmented 3D T1w-FFE sequence with 50 dynamics for a total scan time of 25:52 min. The scan parameters were TR/TE = 16/4.9 ms, turbo factor = 48, SENSE factor 1.5, resolution (freq × phase × slice) = 0.30 × 0.37 × 2.0 mm, tip angle = 80 and two signal averages for a scan time of 31 s/dynamic. Three calibration scans with identical resolution preceded the dynamic sequence with tip angles 50, 100, and 150. The contrast agent was injected after the 5th dynamic scan. The K-trans were calculated using the original Philips software package.

We used two MRI sequences: a T2-weighted (T2W) sequence for anatomical imaging and the validation of brain edema 24 h post-MCAO, and diffusion-weighted imaging (DWI) for measurements of regional apparent diffusion coefficient (ADC) vales during MCAO calculation of ischemic lesion volumes. All two MRI sequences were run to cover the entire brain, in 2-mm thick consecutive coronal slices, with an in-plane field of-view. Other MRI parameters in play were as follows: T2-weighted. Repetition time (TR)/echo time (TE) = 3000/80 ms was used to acquire the T2W turbo spin echo (TSE) sequence. The turbo factor was 14. The in-plane resolution was 0.37 × 0.31 mm, matrix size 192 × 182, with a slice width of 2.0 mm. We acquired 14 slices in the axial plane with zero gaps and four averages after scan completion at 5:18 min.

The diffusion tensor imaging (DTI) sequence served as a multi-shot, stimulated echo acquisition mode (STEAM) planar imaging (EPI) sequence to reduce susceptibility artifacts. The repetition time/mixing-time/echo time (TR/TM/TE) was 1355/15/143 ms. The SENSE parallel imaging factor was 1.3, and the epi (turbo) factor was 19. The *b*-value for obtaining six diffusion gradient encoding directions was 1000 s/mm2. The in-plane resolution used to create seven contiguous 2.0 mm thick slices in the axial plane was 0.50 × 0.51 mm, with matrix size 100 × 82. We were able to obtain six averages from scanning in 8:40 min.

An expert blinded to the experimental procedure carried out image analysis using a Philips software package and an ImageJ software, performed calculations, and analyzed results. The Philips software package (Ingenia, Philips Medical Systems, Best, Netherlands) was used to individual ROI’s for assessment BBB breakdown and to produce a map for the brain images generate quantitative ADC maps, in mm^2^/s, which were subsequently analyzed using the Image J software 1.50i version^1^, described previously (Boyko et al., 2019b; Frank et al., 2019). The viability thresholds used for the identification of every pixel showing irregular ADC features on the slide were 0.53 × 10^–3^mm^2^/s for ADC (Bardutzky et al., 2005).

Calculation of infarcted zone was performed by the Ratios of Ipsilateral and Contralateral Cerebral Hemispheres (RICH) method. The calculation of the lesion volume with the correction for tissue swelling by the RICH technique was done using the following formula (Boyko et al., 2013, 2019b): Corrected infarct size = infarct size × contralateral hemisphere size/ipsilateral hemisphere size. The infarcted brain volume was measured as a percentage of the total brain (Boyko et al., 2013, 2019b).

Calculation of brain edema was performed by the RICH method. The calculation of brain edema by the RICH technique was done by comparing the contralateral and ipsilateral hemispheres, and performed using the following formula: Brain edema = (the volume of right hemisphere – the volume of left hemisphere)/the volume of left hemisphere (Boyko et al., 2019a).

### Statistical Analysis

Statistical analysis was performed with the SPSS 22 package (SPSS Inc., Chicago, IL, United States). The Kolmogorov–Smirnov test was used, considering the number of rats in each group for deciding the appropriate test for the comparisons between the different parameters. For non-parametric data, we used the appropriate tests suitable for non-parametric data. The neurological severity scores are expressed as the median and 25–75 percentile range, and were compared by the Mann–Whitney *U* test. Infarct volume, brain edema and BBB breakdown are expressed as a mean percentage of the contralateral hemisphere ± SEM, or mean Evans blue extravasation index in ng/g of brain tissue ± SEM. and compared by the Mann–Whitney *U* test or *t*-test according to Kolmogorov–Smirnov test and group size. We calculated the correlation between (1) infarct zone (2) brain edema and (3) BBB breakdown assessment by MRI and histological methods. A correlation was also calculated for analyzing the standard curve for measurements of Evans blue extravasation index. Correlations were calculated using the Spearman’s test for non-parametric data or Pearson’s test for parametric data. Criteria for parametric data were (1) normal distribution (The Kolmogorov–Smirnov test was used) (2) *n* > 30 and (3) the data correspond to an interval scale. The various concentration of TTC in brain samples were compared to brain samples with 0% TTC concertation using the Wilcoxon Signed Ranks Test. Results were considered statistically significant when *P* < 0.05, and highly significant when *P* < 0.01.

## Results

### Mortality

The mortality rate in this study was 20.0% for the 60 total rats that underwent MCAO (48 survived). There was a 0% mortality rate in the 36 sham-operated rats.

### Neurological Deficit

Of the 48 rats who survived the MCAO procedure, 6 rats that that did not develop neurologic deficits at 24 h post-MCAO were excluded. The remaining rats who underwent MCAO had significantly impaired neurologic performance, i.e., a higher neurological severity score, compared to controls (*p* < 0.001). The median neurological severity score was significantly higher in these 42 post-MCAO rats compared to the 36 sham-operated rats (3, range 2–4 vs. 0, range 0–0, *p* < 0.001) according to a Mann–Whitney test. The data are measured as a count and expressed as median and 25–75 percentile range.

### Brain Infarct Volume

The infarct zone, measured 24 h after stroke induction, was significantly increased for the 11 rats that underwent MCAO compared to the 10 sham-operated rats using the original technique (8.27% ± 1.78 vs. -0.18% ± 0.75, *U* = 1, *p* < 0.01, *r* = 0.69, according Mann–Whitney test). Using the new technique, there was a significant increase in the brain infarct volume after 24 h in the 19 rats that underwent MCAO compared to the 16 sham-operated rats [7.49% ± 0.82 vs. 0.31% ± 0.48, *t*(28.49) = 7.56, *p* < 0.01, according to independent-samples *t*-test]. There was no significant difference between the original and new technique in measuring infarct volume (Figure 2E). The data is expressed as a mean percentage of the contralateral hemisphere ± SEM.

### Brain Edema

The extent of brain edema, measured 24 h after stroke induction, was significantly increased for the 11 rats that underwent MCAO compared to the 10 sham-operated rats using the original technique (12.53% ± 2.65 vs. 1.54% ± 2.31, *U* = 22, *p* < 0.05, *r* = 0.562, according Mann–Whitney test). Using the new technique, there was a significant increase in the extent of brain edema after 24 h in the 19 rats that underwent MCAO compared to the 16 sham-operated rats [12.31% ± 1.97 vs. 0.64% ± 2.57, *t*(29.37) = 3.61, *p* = 0.01, *d* = 1.23, according to independent-samples *t*-test]. There was no significant difference between the original and new technique in measuring brain edema (Figure 2F). The data is expressed as a mean percentage of the contralateral hemisphere ± SEM.

### BBB Breakdown

The analysis of BBB breakdown (presented in Figures 2G,H), measured by the spectroscopic method 24 h after stroke induction, was significantly increased for the 12 rats that underwent MCAO compared to the 10 sham-operated rats using the original technique (2352 ng/g ± 194 vs. 85 ng/g ± 8, *U* = 0, *p* < 0.01, *r* = 0.92, according Mann–Whitney test). Using the new technique, there was a significant increase in the extent of BBB breakdown after 24 h in the 19 rats that underwent MCAO compared to the 16 sham-operated rats [2235 ng/g ± 253 vs. 94 ng/g ± 9, *t*(18.05) = 8.47 *p* < 0.01, *d* = 2.7, according to independent-samples *t*-test]. There was no significant difference between the original and new technique in measuring brain edema (Figure 2H). The data are measured in ng/g of brain tissue and presented as mean ± SEM.

The BBB breakdown, measured by the evaluation of the scanned brain slices 24 h after stroke induction, was significantly increased for the 12 rats that underwent MCAO compared to the 10 sham-operated rats using the original technique (7.54% ± 0.72 vs. 0.53% ± 0.27, *U* = 0, *p* < 0.01, *r* = 0.88, according Mann–Whitney test; Figure 2B). More specifically, there was a significant difference in BBB breakdown measured by the brain image scanning method in all 6 brain slices in rats post-MCAO compared to the 6 brain slices from the sham-operated rats (Figure 2A). Using the new technique, there was a significant increase in the extent of BBB breakdown after 24 h in the 19 rats that underwent MCAO compared to the 16 sham-operated rats [7.06% ± 0.9 vs. 0.22% ± 0.42, *t*(25.78) = 6.85, *p* < 0.01, *d* = 2.26, according to independent-samples *t*-test]. There was no significant difference between measuring BBB breakdown by spectroscopy compared to the brain image scanning method. The data is expressed as a mean percentage of the contralateral hemisphere ± SEM. Analysis of the Evans blue extravasation index in brain tissue measured by the spectroscopic method and by the evaluation of scanned brain slices showed a high and significant correlation (*r* = 0.833, *p* < 0.01; Figure 2G).

### Evans Blue on the Accuracy of Measuring Infarct Zone

In order to evaluate the effect of Evans blue on the accuracy of measuring infarct zone, we compared the Evans blue extravasation index in scanned brain slices after applying the blue filter. These brain slices were scanned before being homogenized in 1 ml of 50% trichloroacetic acid during the measurement of BBB disruption via the original technique. After the application of the blue filter, there was no significant difference found between rats that underwent MCAO compared to sham-operated rats (Figures 2C,D). The data is expressed as a mean percentage of the contralateral hemisphere ± SEM.

### TTC on the Accuracy of Measuring BBB Permeability

**FIGURE 3:**
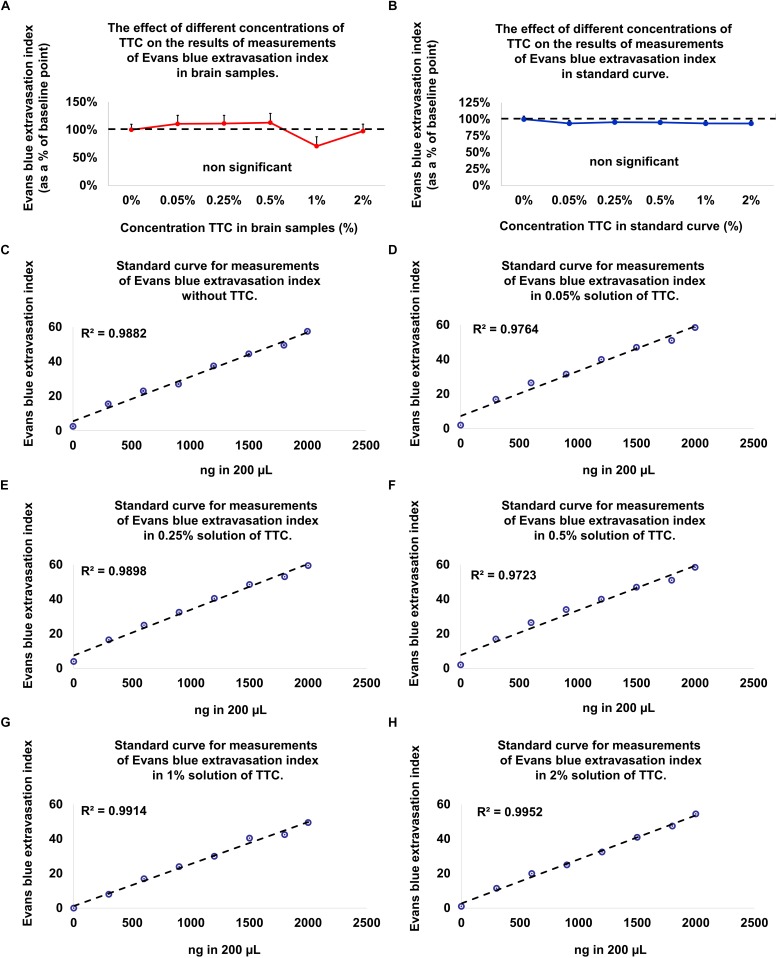
Evolution of the Evans blue standard curve and in brain samples at different TTC concentrations compared to baseline (without TTC). **(A)** There is no difference in the Evans blue extravasation index for the standard curve without TTC compared to brain tissue samples incubated in TTC. Data is presented as a percentage of baseline ± SEM. **(B)** There is no difference in the Evans blue extravasation index for the brain tissue samples without TTC compared to brain tissue samples incubated in TTC. Data is presented as a percentage of baseline ± SEM. **(C–H)** An Evans blue standard curve with concentrations ranging from 0 to 2000 ng in 200 μL DDW and amount of TTC solution (0–2%), equivalent to the content in the brain tissue.

Since the new technique of measuring BBB permeability used brain slices that were preincubated in TTC, we studied the effects of various concentrations of TTC (0, 0.05, 0.35, 0.5, 1, and 2%) on the accuracy of measuring BBB permeability. The Evans blue extravasation index for the brain tissue samples without TTC were not significantly different than the brain tissue samples incubated in TTC at concentrations of 0.05–2% compared to 0% (Figure 3A and Table 1). Creating standard curves for Evans blue with concentrations ranging from 0 to 2000 ng in 200 μL DDW, we found no significant difference between brain samples without TTC and brain samples incubated in TTC at concentrations of 0.05–2% (Figure 3B and Table 2). Furthermore, we found a high correlation in the Evans blue standard curve at each of the incubated TTC concentrations tested (Figures 3C–H).

**TABLE 1:** Evolution of the Evans blue extravasation index in brain samples at different TTC concentrations compared to baseline (brain samples without TTC).

**Groups**	***n* = 96**		
	
	**Mean and SEM%**	**Variability %**	***P*-value**
Measurements of Evans blue extravasation index in brain samples without TTC.	100 ± 10	97	
Measurements of Evans blue extravasation index in brain samples amount 0.05% solution of TTC, equivalent to the content in the brain tissue.	110.6 ± 15.3	135	Non-significant compare to baseline
Measurements of Evans blue extravasation index in brain samples amount 0.25% solution of TTC, equivalent to the content in the brain tissue.	111.3 ± 14.7	129	Non-significant compare to baseline
Measurements of Evans blue extravasation index in brain samples amount 0.5% solution of TTC, equivalent to the content in the brain tissue.	112.9 ± 16.5	144	Non-significant compare to baseline
Measurements of Evans blue extravasation index in brain samples amount 1% solution of TTC, equivalent to the content in the brain tissue.	70.6 ± 16.9	235	Non-significant compare to baseline
Measurements of Evans blue extravasation index in brain samples amount 2% solution of TTC, equivalent to the content in the brain tissue.	97.6 ± 12.5	125	Non-significant compare to baseline

**TABLE 2:** Evolution of the Evans blue standard curve at different TTC concentrations compared to baseline (Evans blue standard curve without TTC).

**Groups**	***n* = 96**			
	
	**Mean and SEM%**	**Variability %**	***R*^2^**	***p*-value**
An Evans blue standard curve was established with concentrations ranging from 0 to 2000 ng in 200 μL DDW	100 ± 4.6	46	*R*^2^ = 0.9988	
An Evans blue standard curve was established with concentrations ranging from 0 to 2000 ng in 200 μL DDW and amount of 0.05% TTC solution, equivalent to the content in the brain tissue.	93.6 ± 4.5	47	*R*^2^ = 0.9764	Non-significant compare to baseline
An Evans blue standard curve was established with concentrations ranging from 0 to 2000 ng in 200 μL DDW and amount of 0.25% TTC solution, equivalent to the content in the brain tissue.	95.4 ± 2.9	29	*R*^2^ = 0.9898	Non-significant compare to baseline
An Evans blue standard curve was established with concentrations ranging from 0 to 2000 ng in 200 μL DDW and amount of 0.5% TTC solution, equivalent to the content in the brain tissue.	95.2 ± 2.2	23	*R*^2^ = 0.9723	Non-significant compare to baseline
An Evans blue standard curve was established with concentrations ranging from 0 to 2000 ng in 200 μL DDW and amount of 1% TTC solution, equivalent to the content in the brain tissue.	93.6 ± 4.5	47	*R*^2^ = 0.9914	Non-significant compare to baseline
An Evans blue standard curve was established with concentrations ranging from 0 to 2000 ng in 200 μL DDW and amount of 2% TTC solution, equivalent to the content in the brain tissue.	93.5 ± 4.5	48	*R*^2^ = 0.9952	Non-significant compare to baseline

### Comparison to MRI Techniques

MRI techniques for assessing neurologic injury post-MCAO was compared to histological techniques. There was a high correlation between the TTC staining, %RICH lesion volume calculation with edema correction assessment, and the ADC-applied MRI assessment of the lesion volume [*r*s(36) = 0.739, *p* < 0.01], 24 h after ischemia onset. There was a moderate correlations between the T2% RICH -MRI assessment of brain edema and %RICH assessment of brain edema in histological methods [*r*s(36) = 0.633, *p* < 0.01], 24 h after ischemia onset. There was a low correlation between the Ktrans -MRI assessment and the Evans blue extravasation index performed by the histological method [*r*s(36) = 0.46, *p* < 0.01], 24 h after ischemia onset (Table 3).

**TABLE 3:** Comparing histological and MRI techniques on assessment of neurologic injury.

	**MRI techniques**		
	
	**Brain Edema**	**K^*trans*^ extravasation**	**ADC**
	**from T2**	**index**	
**Histological method**			
Brain Edema	*R*s = 0.633; *n* = 36		
Evans blue extravasation index		*R*s = 0.46; *n* = 36	
Infarct zone			*R*s = 0.739; *n* = 36

*There was a high correlation between the TTC staining, %RICH lesion volume calculation with edema correction assessment, and the ADC-applied MRI assessment of the lesion volume [*r*s(36) = 0.739, *p* < 0.01], 24 h after ischemia onset. There was a moderate correlations between the T2% RICH -MRI assessment of brain edema and %RICH assessment of brain edema in histological methods [*r*s(36) = 0.633, *p* < 0.01], 24 h after ischemia onset. There was a low correlation between the Ktrans -MRI assessment and the Evans blue extravasation index performed by the histological method [*r*s(36) = 0.46, *p* < 0.01], 24 h after ischemia onset. MRI, Magnetic Resonance Imaging; TTC, 2,3,5-triphenyltetrazolium chloride; RICH, the Ratios of Ipsilateral and Contralateral Cerebral Hemispheres.*

## Discussion

In this study, we describe a novel method of measuring cerebral edema, infarct zone and BBB permeability following MCAO in the same set of brain samples. Our results demonstrate that these parameters of neurological injury following MCAO can accurately be measured in the same set of brain samples.

We compared the original techniques of measuring post-MCAO infarct zone, cerebral edema and BBB breakdown individually on different brain samples, to a new technique of measuring all of these parameters together in a single set of brain samples. For this purpose, we used three groups of rats. Infarct zone and brain edema was measured in group one using the original technique previously described (Kaplan et al., 1991; Joshi et al., 2004; Boyko et al., 2011a, 2013), BBB disruption was measured in the second group using the original technique previously described (Boyko et al., 2012), and all 3 parameters were measured in the third group on a single set of brain samples using a new technique. Both the original and new techniques were able to correctly identify neurological injury following MCAO in all three groups compared to sham-operated controls. Furthermore, there were no significant differences found between the new and old techniques.

We further wanted to evaluate the effect of Evans blue on the accuracy of infarct zone measurements. For these measurements, the ImageJ computer program converts the scan into a black and white image and then uses a threshold function to mask and calculate the pixels that are either black or white. Brain areas dyed with Evans blue, prior to incubation in TTC solution, were found to be categorized as normal (black) tissue. As a result, no difference could be found in the infarct zone between rats that underwent MCAO compared to control sham-operated rats. This feature actually allows a method of evaluating the area of BBB breakdown by scanned images, as previously described (Boyko et al., 2013). Our results suggest a high correlation between measuring BBB via this brain image scanning method compared to a traditional spectrometric method. Adding a blue filer on scanned brain slices to remove the effects of Evans dye allowed the infarct zone to be measured.

Since the new technique of measuring BBB permeability used brain slices that were preincubated in TTC, we studied the effects of various concentrations of TTC (0, 0.05, 0.35, 0.5, 1, and 2%) on the accuracy of measuring BBB permeability. Our protocol requires TTC concentrations of 0.05%; however, the literature describes protocols with concentrations of TTC up to 2% (Popp et al., 2009). Our results suggest that there was no difference in the Evans blue extravasation index between the brain tissue samples without TTC and brain tissue samples incubated in TTC at concentrations of 0.05–2%.

The goal of this study was to evaluate an alternative method of measuring three parameters of neurological injury following MCAO (cerebral edema, infarct zone and BBB permeability) via histologic examination in the same set of brain samples. It’s important to note that this is also possible to do with MRI technology. MRI also avoids euthanasia, which is ethically preferred. MRI is further useful for behavioral studies in post-stroke rats, and we have used this technique in our own laboratory in the past for these experiments (Frank et al., 2019). However, in practice histological methods are much more commonly used and remain the gold standard for assessing brain injury after stroke (Liu et al., 2009). This is likely due to the economic burden and limited access and availability of MRI equipment in most laboratories. The resolution using histological methods are also currently superior to MRI technology, even with high tesla magnets. Here, we compared histological and MRI techniques on assessment of neurologic injury. Due to the fact that the correlation between BBB rupture and cerebral edema, which we obtained by comparing MRI and histological methods, did not reach high levels, we considered that comparing the data obtained using the same methodology (i.e., histological) would be methodologically more accurate. However, a limitation to this study is that the new histological technique was not directly compared to known *in vivo* techniques of assessing neurologic injury, such as MRI.

## Conclusion

This study describes the efficacy of a new technique to evaluate cerebral edema, infarct zone and BBB permeability after MCAO in the same set of brain samples. Our results suggest no difference in histological outcomes using this new method compared to the original method of using different brain samples for each measurement. Statistical analysis did not show an effect of TTC on BBB permeability measurements using spectrometry. Furthermore, the results suggest that the use of a blue filter completely removes the blue color from the scanned brain images and makes it possible to measure the infarct zone and brain edema effectively. We believe that this novel approach is a practical, accurate, cost-efficient, and ethically favorable model for measuring neurological injury after MCAO, and may promote future studies to help better understand and treat stroke.

## Data Availability Statement

The datasets generated for this study are available on request to the corresponding author.

## Ethics Statement

The animal study was reviewed and approved by the Animal Care Committee of Ben-Gurion University of the Negev, Israel and conducted in accordance with the recommendations of the Declaration of Helsinki and Tokyo and the Guidelines for the Use of Experimental Animals of the European Community.

## Author Contributions

RK, DF, BG, and MB: study conception, data collection and analysis, and manuscript writing/editing and final approval. JG, IM, BK, OT, VZ, IS, and AZ: data collection and analysis, and manuscript editing and final approval.

## Conflict of Interest

The authors declare that the research was conducted in the absence of any commercial or financial relationships that could be construed as a potential conflict of interest.

## Footnotes

1 http://rsb.info.nih.gov/ij/
